# P-1950. Updated Epidemiology of Candidemia in the United States, 2015-2024: Using A Large Nation-Wide Electronic Health Record Database

**DOI:** 10.1093/ofid/ofaf695.2118

**Published:** 2026-01-11

**Authors:** María A Pérez-Ardila, Khush Patel, Laila Bekhet, Cesar A Arias, Max W Adelman, Masayuki Nigo

**Affiliations:** Houston Methodist Research Institute, Houston, TX; Houston Methodist Hospital, Houston, Texas; School of Biomedical Informatics, University of Texas Health Science Center at Houston, Houston, Texas; Houston Methodist and Weill Cornell Medical College, Houston, TX; Houston Methodist Hospital, Houston, Texas; Houston Methodist Hospital, Houston, Texas

## Abstract

**Background:**

Candidemia is associated with high morbidity and mortality in hospitalized patients. The *Candida* species responsible for candidemia has shifted over the past decade. While *C. albicans* has been the most common species, drug-resistant species like *C. auris* are emerging. These changes highlight the need for ongoing monitoring of *Candida* epidemiology in the United States (U.S.)

**Figure 1.**
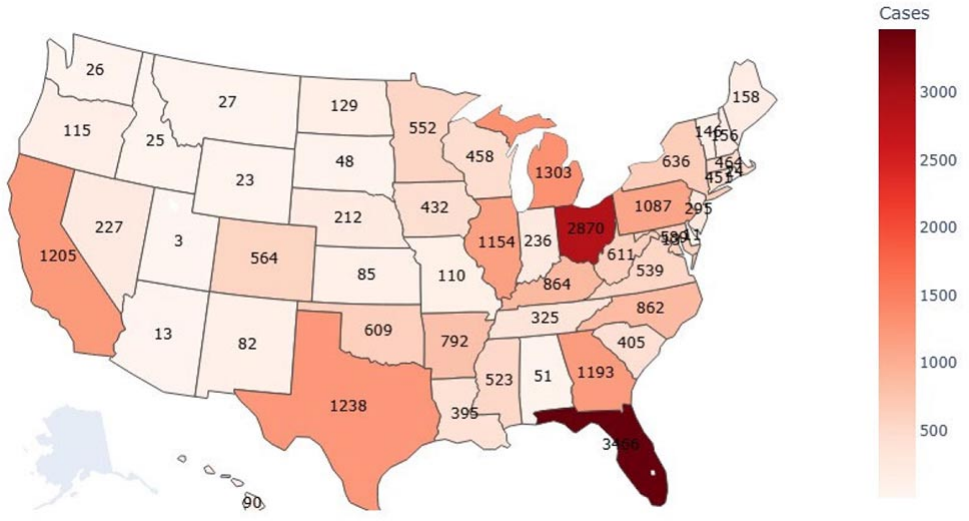
Distribution of candidemia cases that contributed to the study.

**Figure 2.**
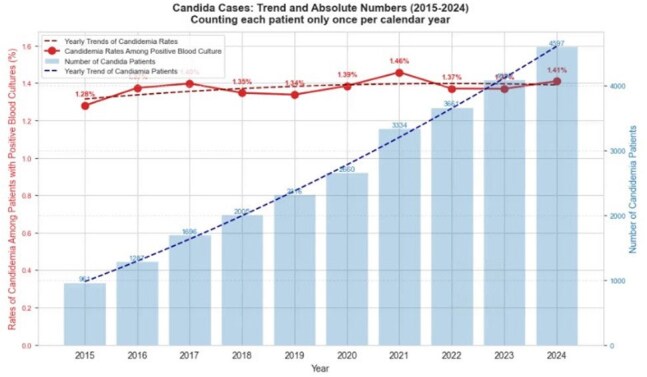
Candidemia incidence rates among positive blood cultures and number of candidemia cases over time

**Methods:**

Retrospective cross-sectional study using data from Epic COSMOS, a de-identified electronic health record dataset (EHR) including 1,626 hospitals and 289 million patients across the U.S. Candidemia cases between 2015-2024 were identified from microbiology susceptibility and molecular results.

**Figure 3.**
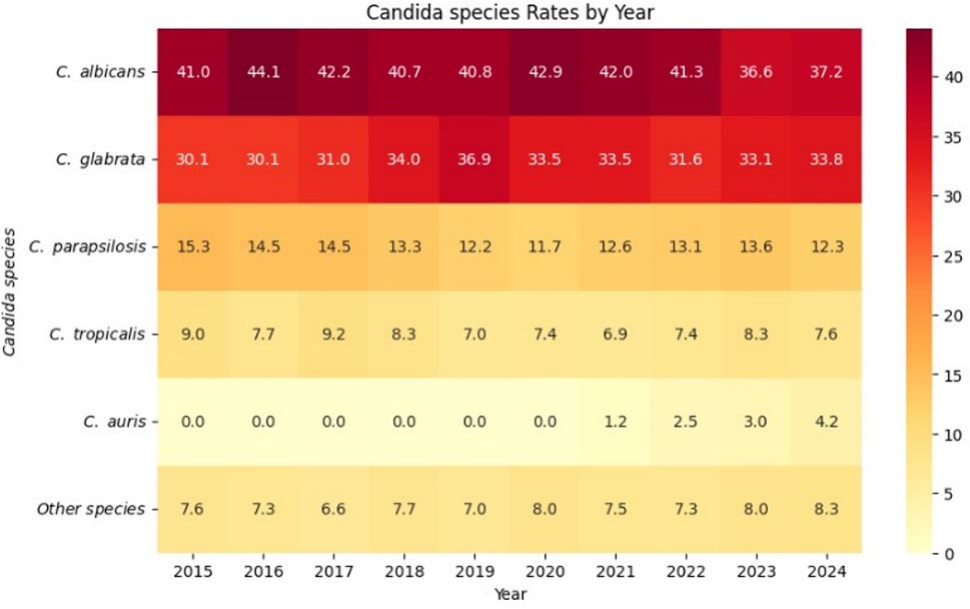
Distribution of Candida species among candidemia over time.

**Figure 4.**
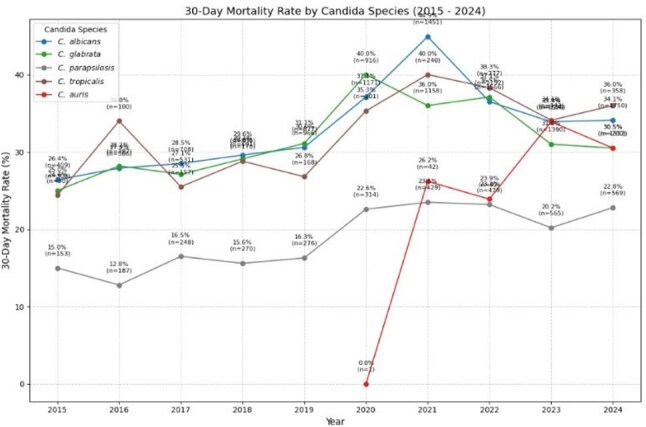
30-day mortality rate associated with candidemia based on Candida species.

**Results:**

We identified 21,961 cases of candidemia among 26,050 unique patients. The median age was 64 years. The cohort was predominantly male (55.7%), with 7.9% identifying as Hispanic and 70% as White, followed by 20.9% African American. Figure 1 shows the geographic distribution of cases contributed to this analysis, with the highest contribution of cases from Florida and Michigan, with fewer from the Northwest. Figure 2 demonstrates a stable incidence of candidemia over time: 1.35% of bloodstream infections were due to *Candida* spp. Among candidemia, while *C. albicans* is the most common species, there was a decline from 41% in 2015 to 37% in 2024. (Figure 3) In contrast, other non-*albicans* species, such as *C. glabrata*, have increased, with *C. auris* emerging in recent years and reaching 4% in 2024. As illustrated in Figure 4, the 30-day mortality varies across species: highest in *C. albicans* and *C. tropicalis*, where mortality exceeds 34%. Noticeably, those mortality rates have declined after a peak during the COVID-19 pandemic. However, *C. auris* mortality has increased since its emergence, reaching 30% in 2024.

**Conclusion:**

This study using a large nationwide de-identified EHR database demonstrated overall stable incidence rates of candidemia. However, among species, non-*albicans* species, including *C. auris*, were gradually increasing over time. This shift in epidemiology along with high mortality rates, especially in *C. auris*, highlights the importance of closely monitoring candidemia epidemiology on a large scale.

**Disclosures:**

All Authors: No reported disclosures

